# Addressing non-communicable diseases and injuries through an equity lens: insights from the Cameroon non-communicable disease and injury (NCDI) Poverty Commission

**DOI:** 10.7189/jogh.16.04266

**Published:** 2026-10-09

**Authors:** Yauba Saidu, Michael Budzi, Hassan Ben Bachire, Diaby Ousmane, Farida Haoua, Mapa Clarisse, Vaissaba Emmanuel, Marie Solange Ndom, Clarence Mbanga, Ismaila Karimu, Mbiydzenyuy Ferdinant Sonyuy, Emmanuel Mensah, Gene Bukhman, Neil Gupta, Basile Njei, Yazan A Al Ajlouni, Olufunke Fasawe, Suzanne Sap, Eugene Sobngwi, Paul Ndom, Samuel Kingue

**Affiliations:** 1 Clinton Health Access Initiative, Yaoundé, Cameroon; 2 Institute for Global Health, University of Siena, Siena, Italy; 3 Department of Health and Health Professions, University of Florida, Gainesville, Florida, USA; 4 Department of Health Promotion, Ministry of Public Health, Yaoundé, Cameroon; 5 Department of Studies and Projects, Ministry of Public Health, Yaoundé, Cameroon; 6 Department of Disease Control, Epidemics and Pandemics, Ministry of Public Health, Yaoundé, Cameroon; 7 Laquintinie General Hospital, Douala, Cameroon; 8 Faculty of Health Sciences, University of Buea, Cameroon; 9 Reconciliation and Development Association (RADA), Bamenda, Cameroon; 10 Center for Integration Science, Brigham & Women’s Hospital, Boston, Massachusetts, USA; 11 Yale University School of Medicine, New Haven, Connecticut, USA; 12 Section of Digestive Diseases, Department of Medicine, Yale University, New Haven, Connecticut, USA; 13 Engelhardt School of Global Health and Bioethics, Euclid University, Bangui, Central African Republic; 14 Veterans Affairs (VA) Connecticut Healthcare, West Haven, Connecticut, USA; 15 Ohio University Heritage College of Osteopathic Medicine, Athens, Ohio, USA; 16 Yale Liver Center, Yale New Haven Health, New Haven, Connecticut, USA; 17 Department of Rehabilitation, Montefiore Medical Center, Bronx, New York, USA; 18 The University of Ebolowa, Ebolowa, Cameroon; 19 Faculty of Medicine and Biomedical Sciences, University of Yaoundé 1, Yaoundé, Cameroon

**Keywords:** non-communicable diseases and injuries, poverty commission, Cameroon

## Abstract

**Background:**

Non-communicable diseases and injuries (NCDIs) constitute a leading cause of premature mortality globally, disproportionately affecting low- and middle-income countries (LMICs). To curb the rising burden of NCDIs in Cameroon, the Ministry of Public Health established the National NCDI Poverty Commission to assess the burden of NCDIs, evaluate investments and expenditures related to NCDI services, and generate evidence to guide national policy formulation and resource prioritisation.

**Methods:**

We synthesised data from the Global Burden of Disease (GBD) 2019 database and conducted a comprehensive literature review of peer-reviewed and grey literature (2005–2022) to characterise the burden and risk factors of NCDIs in Cameroon. Investments and expenditures related to NCDIs were extracted from the World Health Organization Global Health Expenditure Database and national planning documents. A two-phase priority-setting process was applied to rank NCDI conditions and identify cost-effective, context-appropriate interventions through quantitative scoring and expert consensus.

**Results:**

NCDIs accounted for 43% of all deaths in 2019, with nearly 29% occurring among individuals aged <40 years. Disability-adjusted life years (DALYs) from NCDIs have risen steadily since 1990, with almost two-thirds (61%) attributable to conditions beyond the ‘big four’ disease categories of diabetes, neoplasms, chronic respiratory diseases, and cardiovascular diseases. Over half (51%) of NCDI-related DALYs in Cameroon were not attributable to quantified behavioural or metabolic risk factors in the GBD framework. Despite this high burden, only 6% of disease prevention funding was directed to NCDIs between 2016 and 2020. The Commission identified 49 priority NCDI conditions and 123 interventions for phased implementation across community, primary, and hospital platforms.

**Conclusions:**

NCDIs are a major public health burden in Cameroon, heavily impacting the ‘economically productive’ age group. Rebalancing financing and integrating a prioritised package of NCDI services into Universal Health Coverage (UHC) would be essential to reduce premature mortality and financial hardship.

Non-communicable diseases and injuries (NCDIs) are the principal causes of global mortality, significantly impacting health and development [1]. Globally, the combined burden of non-communicable diseases and injuries (NCDIs), encompassing non-communicable diseases as well as unintentional and intentional injuries, now exceeds the aggregate mortality burden from HIV, tuberculosis, and maternal causes, particularly among populations aged <40 years [2]. The burden of NCDIs further exacerbates health inequality as it disproportionately affects socioeconomically disadvantaged individuals, particularly those in low and middle-income countries (LMICs) [3], where it imposes substantial economic hardship on households already experiencing high levels of healthcare expenses annually [4].

NCDI Poverty Commissions have emerged as pivotal entities in the global fight against NCDIs, particularly in LMICs. Typically, these commissions focus on the burden of NCDIs, guiding strategic resource allocation, and informing policy formulation to address these health challenges effectively [5]. In several LMICs, such commissions have played crucial roles in raising awareness, strengthening healthcare infrastructure, and improving access to essential NCDI services. They have also facilitated the prioritisation of interventions, guided resource allocation, and supported the implementation of evidence-based policies to reduce the NCDI burden [6].

Cameroon presented a compelling case for such an initiative due to its rising NCDI burden, notable gaps in policy implementation, and ongoing efforts to advance equitable healthcare access. Although services and policies targeting major NCDI risk factors, such as tobacco use, harmful alcohol consumption, and sedentary lifestyles, are in place, there are notable gaps in policy formulation and implementation for addressing unhealthy dietary habits [7]. In response, the Ministry of Public Health established the National NCDI Poverty Commission, mandating it to evaluate the impact of NCDIs, assess investments in NCDI services, and inform national policy decisions. Building on the experiences of similar commissions in other countries, the Cameroon commission sought to adapt best practices and implement targeted interventions to enhance healthcare equity. Through collaborative efforts and evidence-based approaches, the commission intended to make substantial strides in mitigating the impact of NCDIs and advancing the country's healthcare landscape towards improved well-being and sustainable development.

Cameroon’s NCDI Poverty Commission initially comprised a multidisciplinary group of 22 experts from diverse institutions – including civil society, clinical practice, and academia – working in collaboration with the NCDI Poverty Network at Brigham & Women’s Hospital. The Commission aimed to conduct a situational analysis, prioritise resources for NCDIs, utilise existing data to inform policies, and identify cost-effective interventions to advance Universal Health Coverage (UHC) in line with the national health sector strategy [8]. This paper presents insights from the Cameroon NCDI Poverty Commission’s assessment to characterise the burden of NCDIs in a Central African context where such data are scarce, and documents the priority-setting process used to identify priority NCDI conditions and interventions.

## METHODS

### Study setting

Cameroon is a Central African country, spanning from the Gulf of Guinea to the Lake Chad Basin and bordered by Nigeria, Chad, the Central African Republic, Gabon, Congo, and Equatorial Guinea. It has an estimated population of about 30 million, with approximately 40% of the population living below the poverty line [9]. Life expectancy is 62 years for males and 66 years for females [10]. The health system is organised into three tiers – central, intermediate, and peripheral – overseen by the Ministry of Public Health [8].

### Study procedures

#### NCDI burden assessment

To estimate the burden of NCDIs, two complementary sources of information were used. The Global Burden of Disease (GBD) 2019 database, developed by the Institute for Health Metrics and Evaluation (IHME) [1]. This database provides standardised, comparable estimates of morbidity and mortality across 204 countries. A comprehensive literature review of peer-reviewed publications and grey literature on NCDIs in Cameroon to complement and contextualise GBD findings and identify research gaps.

#### GBD 2019 data extraction and analysis

Data on NCDIs were extracted from the GBD 2019 database [1] focused on Cameroon-specific estimates for 2019, and included the following key variables: mortality and cause-specific death estimates; disability-adjusted life years (DALYs); years of life lost (YLL), Year lived with disability (YLD). NCDIs were defined according to the NCDI Poverty Network framework, comprising two GBD Level 1 categories. Non-communicable diseases and injuries data for Cameroon were extracted using the GBD Results Tool (Institute for Health Metrics and Evaluation, University of Washington, Seattle, WA, USA). In line with the Journal of Global Health's Guidelines for Reporting Analyses of Big Data Repositories Open to the Public (GRABDROP) [11], we have completed the GRABDROP reporting checklist, provided in the **Online Supplementary Document**.

Cause-specific mortality and morbidity data were analysed descriptively using Microsoft Excel 365 (Microsoft Corporation, Redmond, WA, USA). Mortality rates were calculated as the number of deaths per total population, and proportions were computed by major disease category and by age group, with particular focus on individuals under 40 years to assess age-specific impact. For morbidity, DALYs were analysed by cause and age group to highlight high-burden conditions and quantify cumulative national implications. Trends in DALYs from communicable diseases, NCDs, and injuries were compared for the period 1990–2019 to evaluate transitions in disease burden. All GBD 2019 estimates include 95% uncertainty intervals (UIs) reflecting uncertainty in input data, model parameters, and estimation processes. Throughout this analysis, we report point estimates (mean values).

#### Comprehensive literature review

An exhaustive literature review was conducted to complement GBD findings with locally available evidence on NCDIs in Cameroon. The review covered publications between 1 January 2005 and 31 August 2022, retrieved from PubMed/MEDLINE, Embase, and Google Scholar.

A generic search strategy was developed and adapted for each database to account for differences in indexing and search fields. Searches combined geographic and disease-related terms using Boolean operators. The general structure was: (‘Cameroon’) AND (‘non-communicable diseases’ OR ‘chronic diseases’ OR ‘cardiovascular diseases’ OR ‘diabetes’ OR ‘cancer’ OR ‘respiratory diseases’ OR ‘mental health’ OR ‘neurological disorders’ OR ‘injuries’). Searches were limited to human studies published in English or French between 2005 and 2022. Reference lists of key studies were screened to identify additional relevant articles.

Studies were included if they: focused on one or more NCDI conditions in Cameroon, reported quantitative or qualitative data on prevalence, morbidity, mortality, or risk factors; included at least 100 participants (for quantitative studies); and were published in peer-reviewed journals. Studies were excluded if they were laboratory-based and lacked population relevance, were single case reports, were editorials, or were non-peer-reviewed commentaries. A total of 3,021 records were identified through database searches (PubMed: 1,456; Embase: 982; Google Scholar: 583). After removing 1,254 duplicates, 1,767 unique records were screened by title and abstract. Following full-text review, 391 articles met the inclusion criteria and were included in the synthesis.

To capture evidence beyond peer-reviewed publications, grey literature and institutional sources were also reviewed. These included national policy documents, official reports, and repositories of the World Health Organization (WHO) Regional Office. Other key national references included the National Health Development Plan (2016–2020), the National Health Sector Strategy (2016–2027), the National Cancer Control Strategy (2020–2024) [8,11,12], and other Ministry of Health publications relevant to NCDI prevention and control.

#### NCDI financial landscape analysis

The financial landscape for NCDIs in Cameroon was assessed using secondary data from the WHO Global Health Expenditure Database (GHED) [12] and national planning documents, notably the National Health Development Plan (2016–2020) and the Health Sector Strategy (2016–2027), which provided data on national and external health financing patterns and expenditure allocations. Estimates of prevention financing were derived from planned budget allocations within national health-sector programmatic lines, drawing on the Health Sector Strategy (2016–2027). Budget data combined government and donor resources and focused on Component 2 (Disease Prevention). Within this component, allocations were categorised by disease area to estimate the share directed toward non-communicable diseases and injuries (NCDIs). Data were extracted for 2019, expressed in Central African CFA Francs (XAF), and analysed in nominal terms. Variables of interest included general government health expenditure as a percentage of total health expenditure (THE), out-of-pocket expenditure as a percentage of THE, external health expenditure as a percentage of THE, voluntary and private insurance expenditure as a percentage of THE, and disease-prevention budget allocations by category (infectious diseases, NCDs, and others).

#### Priority setting exercise

The priority setting exercise was implemented in two sequential phases: definition and ranking of priority NCDI conditions, and identification of priority health interventions for each condition. Both phases combined quantitative analysis with deliberative expert review and consensus validation.

In phase one, NCDI conditions from the GBD database were evaluated and scored across four quantitative criteria – burden, severity, disability, and equity – inspired by work from similar commissions [6]: Burden: Burden (DALYs): Conditions were grouped by DALY range and assigned scores from 1 (lowest burden) to 5 (highest burden). Severity (YLL per death): The ratio of Years of Life Lost (YLL) to deaths was used as a mortality-weighted measure of premature death. Conditions were grouped and scored from 1 to 5 based on thresholds ranging from <0.01 (lowest) to >1.50 (highest). Disability (YLD per case): The ratio of Years Lived with Disability (YLD) to the number of cases captured morbidity severity. Conditions were classified into four groups and scored from 1 to 4, with ratios >1 receiving the highest score. Equity: This criterion captured the poverty dimension by comparing the DALY per capita with that of high-income countries. Ratios were grouped into three ranges and scored from 1 (lowest inequity) to 3 (highest inequity). Equity was assessed by comparing Cameroon’s DALY burden with that of high-income countries, defined according to the World Bank income classification. Population-weighted, age-standardised DALY rates per 100,000 from the Global Burden of Disease (GBD) 2019 high-income country aggregate were used as the comparator.

The scoring thresholds and scale ranges were selected pragmatically to stratify conditions across wide distributions of GBD metrics and facilitate relative ranking, rather than to represent clinically meaningful cut-offs. Scores from all four criteria were summed up to obtain a composite priority score for each condition. The conditions were then ranked from highest to lowest composite score. The top 100 ranked conditions were further evaluated using three additional qualitative criteria: Public health importance (national burden and social relevance), Feasibility of health-sector intervention, and Alignment with national policy frameworks. Conditions meeting at least two of these qualitative criteria were retained. The commission also reviewed lower-ranked conditions, including those with strong equity or feasibility relevance that may have been underrepresented by quantitative scoring (*e.g.* mental health and childhood NCDs). Quantitative scores were therefore used as an initial ordering tool and were complemented by structured qualitative review and expert consensus to inform final prioritisation.

In phase two, priority interventions were identified using the FairChoices DCP Analytics Tool (Version 2.1) – a web-based modelling platform developed by the University of Bergen (Bergen, Norway), University of Washington (Seattle, WA, USA), and the Centre for Integration Science in Global Health Equity (Boston, MA, USA) [13]. This tool synthesises evidence from Disease Control Priorities, 3rd Edition (DCP3) and models health gains under budget constraints using country-specific parameters. The tool’s budget space was parameterised with Cameroon-specific financing inputs: baseline and target government, donor, out-of-pocket, and insurance shares; health-expenditure-to- Gross Domestic Product (GDP) ratios; and GDP growth projections. The model then generated feasible benefit packages under the implied resource paths. Interventions were screened with three quantitative filters pre-specified by the Commission: 10-year DALYs gained >20,000, baseline coverage ≥30%; and annual population in need >50,000. Interventions meeting any of these filters were shortlisted for further consideration. Following the model filter, the task force conducted a structured expert review to add context-critical interventions that did not pass the quantitative screen but had high strategic value (*e.g.* equity, feasibility, readiness).

## RESULTS

### Burden of NCDIs in Cameroon

Collectively, NCDIs accounted for to 43% of all deaths in Cameroon in 2019, with 35% resulting from NCDs and 8% from injuries. Approximately 29% of these fatalities occurred in individuals under the age of 40. Four disease categories accounted for 57% of all annual NCDI-related mortality in Cameroon. These included, cardiovascular diseases, like hypertension and stroke, (29%), neoplasms (16%), and diabetes and chronic kidney diseases (11%) (**Figure 1**).

**Figure 1:**
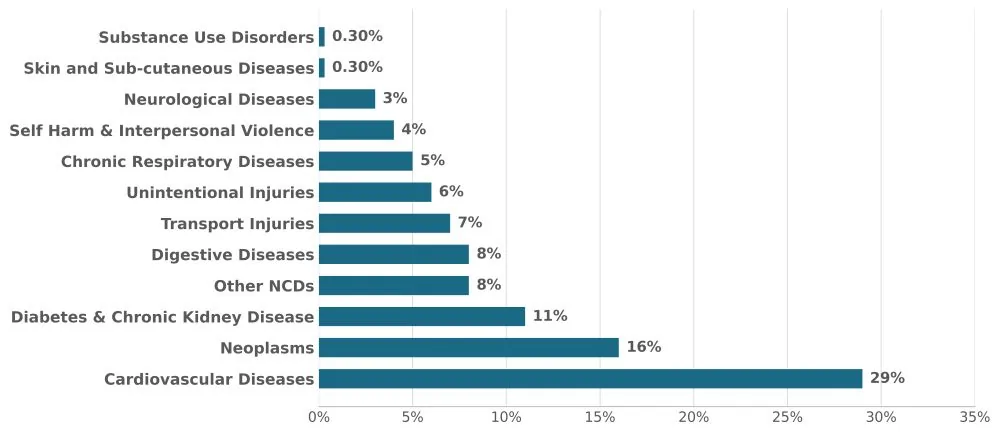
Causes of non-communicable diseases and injuries mortality in Cameroon (GBD 2019).

While the DALYs lost to infectious diseases has witnessed a steady decline over the past 3 decades, DALYs lost to NCDs in Cameroon has recorded a steady increase, since 1990 with a 10% growth by 2019. About two thirds (61%) of all DALYs from NCDIs were attributed to conditions other than the four major categories (the big four) highlighted in the WHO Global Action Plan (GAPs) (**Figure 2**). In the under-40 age group, NCDIs with the highest DALYs were mental health disorders, neurological disorders, and transport-related and unintentional injuries. Cumulatively, injuries (transport injuries, self-harm and interpersonal violence and unintentional injuries) contribute to approximately one fifth (19%) of the national NCDI DALY in Cameroon.

**Figure 2:**
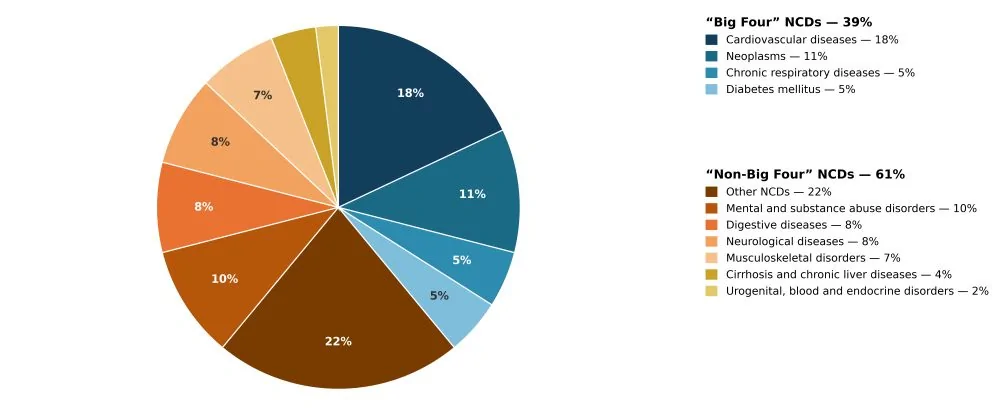
DALY burden attributed to non-communicable diseases and injuries (NCDs) in Cameroon in 2019, proportion attributed to the ‘big four’ *vs.* ‘Non-Big Four’ NCDs (GBD 2019). DALY – disability-adjusted life years, NCDs – non-communicable diseases and injuries.

In the NCDI assessment, we identified both classical modifiable and non-modifiable risk factors. The non-modifiable risk factors included mainly age below 40 years and sex (male or female), while the modifiable factors included metabolic and behavioural factors such as obesity, sedentary lifestyle, dietary patterns, tobacco use, and alcohol misuse. Despite these, over half (51%) of NCDI-related DALYs in Cameroon were not attributable to quantified behavioural or metabolic risk factors.

### NCDI financial landscape analysis

Healthcare financing in Cameroon is primarily characterised by private funding sources, with direct out-of-pocket (OOP) payments accounting for about 73% of total health expenditure. In 2019, current health expenditure represented 4% of GDP, while government health spending accounted for 11% of total health expenditure. A deeper examination showed that only 6% of the disease-prevention budget was allocated to NCDIs between 2016 and 2020. This allocation sharply contrasted the nearly two-thirds (64%) of funds allocated for the prevention of infectious diseases and the 19% for vaccine-preventable diseases and other public health interventions (**Figure 3**). Expectedly, further efforts to obtain holistic NCDI expenditure were unsuccessful, partly due to a lack of systematic documentation of these data points[14,15].

**Figure 3:**
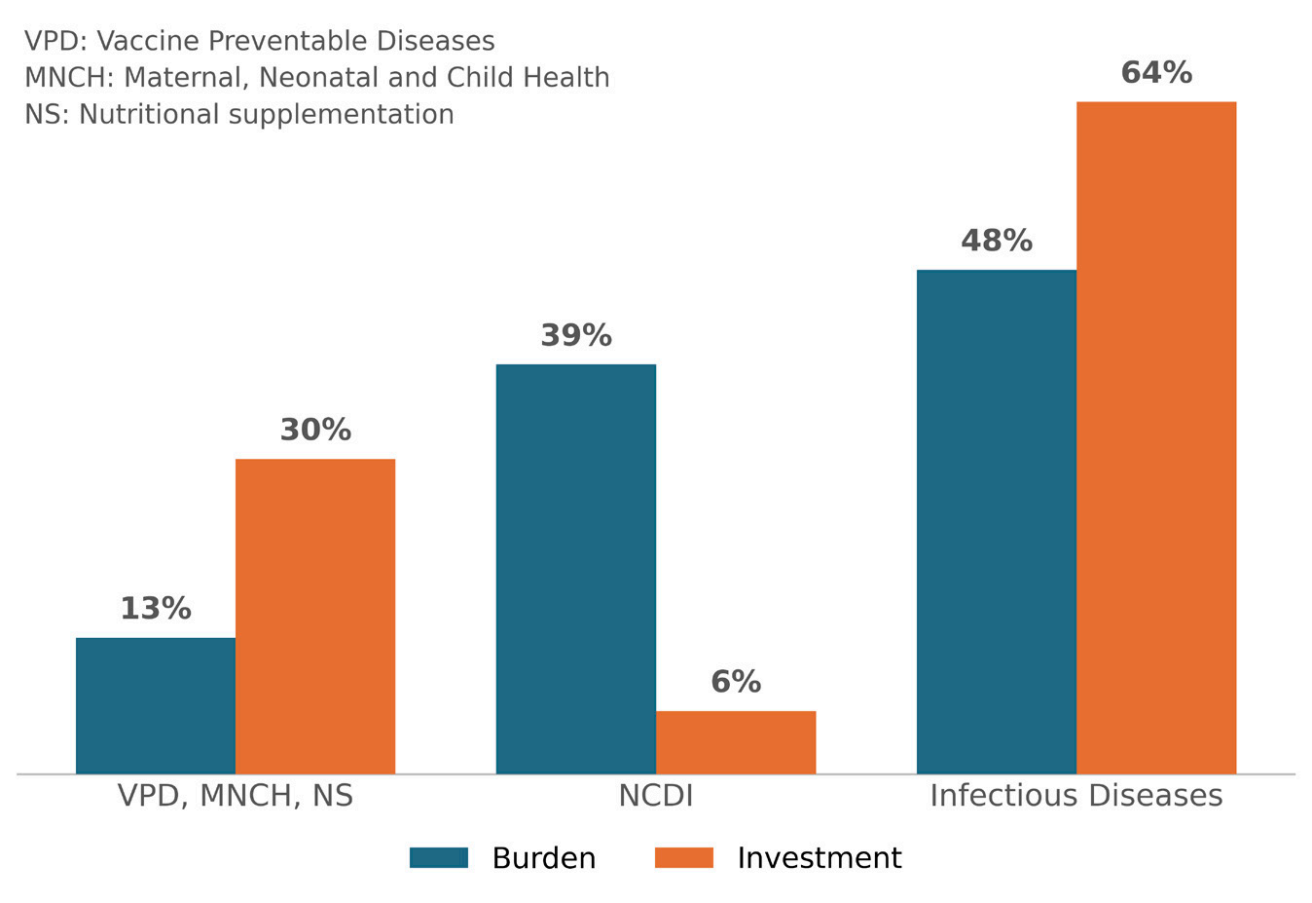
Funding distribution between infectious diseases and non-communicable diseases and injuries.

### Priority NCDI conditions and interventions

Overall, 49 conditions were prioritised based on their importance, the feasibility of implementing interventions for these conditions in Cameroon, and their alignment with national policies (**Table 1**). Most of the prioritised conditions fall under 4 major disease categories, namely cardiovascular (6/49), neoplasms (16/49), and mental disorders (4/49).

**Table 1:** Prioritised NCDI conditions in Cameroon

Disease category	Prioritised conditions	Number of conditions
Neoplasms (adults)	Cervical, brain & CNS, NHL, stomach, tracheal/bronchus/lung, breast, colorectal, Hodgkin lymphoma, nasopharynx, prostate, oesophageal, liver	12
Cardiovascular diseases	Stroke, cardiomyopathy & myocarditis, hypertensive heart disease, rheumatic heart disease, ischemic heart disease	5
Neoplasms (paediatrics)	Acute lymphoid leukaemia, Burkitt’s lymphoma, nephroblastoma (Wilm’s tumour), retinoblastoma and soft tissue sarcoma	5
Mental disorders	Major depressive disorder, anxiety disorders, substance use disorders, bipolar disorders	4
Digestive disorders	Oral disorders, peptic ulcer disease, obesity	3
Injuries	RTA, violence, poisoning	3
Musculoskeletal disorders	Low back pain, osteoarthritis, rheumatoid arthritis	3
Surgical conditions	Appendicitis, inguinal/femoral/abdominal hernia, genital prolapse	3
Congenital disorders	Sickle cell disease, congenital heart anomalies	2
Neurologic disorders	Epilepsy, migraine	2
Renal disorders	Chronic kidney disease, acute kidney injury	2
Respiratory diseases	Asthma, chronic obstructive pulmonary disease	2
Sense organs disorders	Hearing loss, vision loss	2
Endocrine disorders	diabetes mellitus	1

CNS – central nervous system, NCDI – non-communicable diseases and injuries, NHL – non-Hodgkin’s lymphoma, RTA – road traffic accident

A total of 15 interventions were identified for mental health, 14 for cardiovascular diseases and 14 for surgical conditions amongst others, together with their baseline coverage, target coverage and the health-system tiers at which they would be implemented (Table 2). The baseline coverage for the identified interventions varies from as low as 1% for screening for some mental health conditions to about 50% for long term management of epilepsy, a neurological disorder characterising by fainting. Of the identified interventions, over 16% would need to be implemented at community level, 34% at health centre level, 16% at district hospitals and 33% at reference hospitals. To reach UHC at each level, the target coverage should range from 70%–90% at the community level, 60%–80% at the health centre level, 50%–70% at the district level and 40%–60% at the reference hospitals.

**Table 2:** Interventions per disease condition

SN	Interventions	Baseline coverage	Target coverage	Platform
**Mental health interventions**				
1	Tobacco cessation counselling (including nicotine agonist treatment)	31%	80%	Health centre
2	Psychosocial support and centrally acting stimulant (CAS) medication	2%	50%	Health centre
3	Intensive psychosocial treatment and anti-depressant medication for anxiety disorders (moderate-severe cases)	2%	60%	Reference hospital
4	Alcohol use disorders, opportunistic screening, and brief intervention	1%	50%	Health centre
5	Basic psychosocial treatment & anti-depressant medication for anxiety disorders (mod. cases)	2%	80%	Health centre
6	Intensive psychosocial treatment and antidepressant medication of recurrent moderate-severe cases on an epi- sodic/maintenance basis	2%	60%	Reference hospital
7	Opioid Agonist Treatment (OAT) and psychosocial support*	1%	60%	Community
8	Intensive psychosocial treatment and antidepressant medication of first episode severe cases	2%	60%	Reference hospital
9	Basic psychosocial treatment for anxiety disorders (mild cases)	4%	60%	Health centre
10	Drug use disorders, opportunistic screening, and brief intervention	1%	60%	Health centre
11	Basic psychosocial treatment, advice, & follow up for bipolar disorder, mood-stabilising medication	4%	70%	Health centre
12	Basic psychosocial treatment for mild depression	2%	60%	Health centre
13	Basic psychosocial treatment, advice, and follow-up for posttraumatic stress disorder (PTSD)	4%	50%	Health centre
14	Basic psychosocial support and anti-psychotic medication	2%	60%	Health centre
15	Screening of mental disorders in targeted population	2%	50%	Community
**Cancer interventions**				
1	Cryotherapy or loop electrosurgical excision procedure (LEEP)	20%	60%	District hospital
2	Treatment of early-stage cervical cancer: stage I & stage Il	20%	90%	Reference hospital
3	Human papillomavirus (HPV) immunisation	20%	80%	Community
4	Organised screening for cervical cancer: VIA ± HPV 5- yearly for females aged 30–70	20%	90%	Health centre
5	Palliative care for late-stage cervical cancer stage III & stage IV	20%	60%	Reference hospital
6	Confirmatory breast cancer diagnostics and staging	20%	90%	Reference hospital
7	Treatment of Hodgkin lymphoma	8%	80%	Reference hospital
8	Palliative care for late-stage colorectal cancer: stage III & stage IV	20%	60%	Reference hospital
9	Treatment of early-stage colorectal cancer: stage I and stage ll	20%	80%	Reference hospital
10	Treatment of early-stage breast cancer: stage I & stage Il	20%	80%	Reference hospital
11	Education on early warning signs of common & curable paediatric cancers	25%	90%	Community
12	Treatment of Wilms tumour	8%	80%	Reference hospital
13	Treatment of non-Hodgkin lymphoma	8%	80%	Reference hospital
14	Treatment of acute lymphoblastic leukaemia	8%	80%	Reference hospital
**Cardiovascular disease interventions**				
1	Management of acute coronary syndromes (aspirin, unfractionated heparin, thrombolytics...)	31%	70%	Reference hospital
2	Management of chronic heart failure, diuretics, beta-blockers, ACEI and anta-mineralocorticoids	31%	80%	Reference hospital
3	Secondary prevention of stroke	31%	80%	Health centre
4	Management of acute heart failure (diuretics, oxygen, after- load reduction, medication optimisation)	31%	80%	District hospital
5	Secondary prevention of ischemic heart disease (IHD)	31%	80%	Health centre
6	CVD, primary prevention with absolute CVD risk >30% (antihypertensives, statins)	31%	80%	Health centre
7	Aspirin for all cases of high-risk chest pain	31%	80%	Reference hospital
8	Heparin-revascularisation treatment of acute limb ischemia	31%	60%	District hospital
9	Secondary prophylaxis with penicillin for rheumatic fever or known rheumatic heart disease	31%	80%	Health centre
10	Rheumatic heart disease, cardiac surgery	31%	40%	Reference Hospital
11	CVD targeted screening at facility	31%	80%	Community
12	CVD risk prevention and screening in community	31%	80%	Community
13	Treatment of acute pharyngitis in children to prevent rheumatic heart disease	31%	90%	Community
14	Rehabilitation of stroke patients	31%	90%	Reference hospital
**Surgical interventions**				
1	Colostomy due to colon cancer	3%	80%	District hospital
2	Surgical repair of cleft lip and cleft palate	3%	90%	Reference hospital
3	Surgical repair of clubfoot	3%	50%	Reference hospital
4	Surgical management of anorectal malformation	3%	50%	Reference hospital
5	Management of bowel obstruction	3%	50%	Reference hospital
6	Treatment of caries	3%	70%	Health centre
7	Surgery of filarial hydrocele	3%	90%	District hospital
8	Surgery of female genital mutilation complications	50%	90%	Health centre
9	Urinary catheterisation, suprapubic cystostomy	3%	80%	District hospital
10	Hernia repair	3%	80%	District hospital
11	Management of osteomyelitis, including surgical debridement for refractory cases	3%	80%	District hospital
12	Drainage of dental abscess	3%	80%	Health centre
13	Dental extraction	3%	60%	Health centre
14	Appendectomy	3%	90%	District hospital
**Diabetes interventions**				
1	Diabetes opportunistic screening	31%	80%	Health centre
2	Diabetes type 1 insulin	31%	80%	Health centre
3	Diabetes type 2, antidiabetic drugs and insulin	31%	80%	Health centre
4	Screening of diabetes during pregnancy	31%	80%	Health centre
5	Diabetes self-management education	20%	80%	Community
**Neurological Interventions**				
1	Drugs for Parkinson disease (levodopa)	8%	50%	Reference hospital
2	Stabilisation of acute seizures	50%	90%	District hospital
3	Long term management of epilepsy	50%	80%	Health centre
4	Diagnosis of epilepsy	50%	90%	Health centre
5	Diagnostics of Parkinson disease	8%	40%	Reference hospital
**Respiratory disease interventions**				
1	Chronic respiratory disease, vaccination	8%	60%	Health centre
2	Emergency care of severe asthma and COPD (ventilator| Bi-PAP)	8%	70%	Reference hospital
3	Asthma & COPD emergency care (steroids| inhalers| antibiotics| oxygen)	8%	80%	Reference hospital
4	Management of stable COPD (inhalators| anticholinergic agent| smoking cessation	8%	70%	District hospital
5	Management of stable asthma (inhalators| steroids| theophylline	8%	80%	Health centre
**Sense organs interventions**				
1	Retinopathy screening and photocoagulation	31%	80%	District hospital
2	Surgery for trachomatous trichiasis	3%	80%	Reference hospital
3	Glasses for vision problems	20%	60%	Reference hospital
4	Cataract extraction and insertion of intraocular lens	3%	80%	Reference hospital
**Kidney disease interventions**				
1	Chronic Kidney Disease (CKD) risk prevention & screening in the community	8%	60%	Community
2	Albuminuric kidney disease, screening of diabetic patients and management	31%	80%	Health centre
3	Treatment of hypertension in kidney disease, with use of ACEi or ARBs in albuminuric kidney disease	20%	80%	District hospital
**Musculoskeletal disease interventions**				
1	Combination therapy for moderate to severe rheumatoid arthritis, low dose corticosteroids, folic acid supplementation, DMARDs	8%	80%	District Hospital
2	Primary prevention of osteoporosis in high-risk individuals (physical activity, Ca, & vitamin D supplementation)	8%	80%	Health centre
**Cross cutting interventions**				
1	Integrated management of childhood illness	48%	80%	Health centre
2	School based education on sexual health, nutrition, and healthy lifestyle	33%	80%	Community
3	Early childhood development rehabilitation	8%	80%	Community
4	Life skills training in schools to build social & emotional competencies for children & adolescents	33%	80%	Community
5	Education of school children on oral health	33%	80%	Community
6	Mass media messages on sexual & reproductive health & mental health for adolescents	33%	80%	Population based health interventions

ARB – angiotensin II receptor blocker, ACEI – angiotensin-converting enzyme inhibitor, Bi-PAP – bilevel positive airway pressure, COPD – chronic obstructive pulmonary disease, CKD – chronic kidney disease, CVD – cardiovascular disease, DMARDs – disease-modifying antirheumatic drugs, IHD – ischaemic heart disease, VIA – visual inspection with acetic acid

## DISCUSSION

The findings of the Cameroon NCDI Poverty Commission reveal that NCDIs constitute a substantial part of the country’s overall disease burden, particularly affecting individuals under 40 years of age to a greater extent than previously recognised. The spectrum of NCDI conditions is broad, with a significant share of DALYs arising from conditions beyond the traditionally emphasised ‘big four’ diseases (cardiovascular diseases, cancer, diabetes, and chronic respiratory diseases). These include neurological disorders, unintentional injuries, transport-related incidents, and mental health conditions. Mental health emerged as a significant contributor to disease burden, compounding the effects of cardiovascular diseases and neoplasms. The COVID-19 pandemic further intensified these challenges by disrupting chronic disease care and exposing gaps in mental health service provision [16].

Contrary to the standard narrative that metabolic and behavioural factors like obesity, sedentary lifestyles, diet, smoking, and alcohol misuse are the primary drivers of NCDIs [17]. Data from Cameroon suggest that these factors account for less than half of the DALYs among individuals under 40. This pattern highlights the complexity of NCDIs in younger populations and reflects residual burden not attributable to measured behavioural or metabolic risks within the GBD framework. While this residual burden does not imply absence of causation, it points to the potential importance of other structural and social determinants not fully captured in current risk attribution models, including structural and social factors such as poverty, inadequate infrastructure, and limited access to essential health services. Accordingly, while interventions targeting modifiable risk factors remain essential, complementary strategies that address broader contextual and poverty-related determinants are likely necessary to achieve sustained reductions in the NCDI burden in Cameroon.

Cameroon ranks 151st out of 193 countries in the 2021 Human Development Index (HDI), according to the United Nations Development Programme (UNDP), with more than half of the population (55%) living in poverty and over a third (38%) experiencing severe deprivation [18]. Poverty is particularly acute in the Far North and Eastern regions, where recurrent floods, droughts, and underdevelopment further exacerbate health risks and restrict access to care [19]. These socioeconomic disparities have profound implications for NCDI distribution and control. Data from a decade-long study on systolic blood pressure (SBP) in Cameroon showed a more than 2-fold rise in blood pressure among rural populations compared to urban dwellers – rural women experienced an average increase of +18.2 mm Hg *vs.* +8.1 mm Hg among urban women, and rural men experienced +18.8 mm Hg *vs.* +6.5 mm Hg in urban counterparts [20]. These findings illustrate how poverty and environmental adversity, rather than behavioural factors alone, may drive the growing NCD burden in rural settings. As such, comprehensively addressing this diverse range of determinants would be crucial in reducing the burden of NCDIs and enhancing health outcomes across all age groups in the country.

The Commission’s analysis also highlights the persistent inequities in healthcare financing. Health spending in Cameroon remains predominantly private and out-of-pocket, with government expenditure well below the 15% Abuja Declaration target [21]. Moreover, existing allocations inadequately prioritise NCDIs despite their substantial contribution to morbidity and mortality. This pattern mirrors experiences in other West and Central African countries, where limited domestic funding and the absence of dedicated NCDI budget lines have constrained the implementation of effective prevention and control strategies. While the government has made notable progress in integrating care and treatment for some NCDIs (*e.g.* chronic kidney disease) into its universal health package, the overall investments seem inadequate to mitigate catastrophic expenditures from NCDIs [15].

The Cameroon NCDI Poverty Commission has expanded beyond the WHO GAP framework [22] to encompass additional conditions, such as injuries, palliative care, mental health, renal, endocrine, neurological, congenital, musculoskeletal, and surgical disorders, amongst others. The commission’s findings highlight the existence of policies targeting major NCD risk factors such as tobacco use, harmful alcohol consumption, and sedentary lifestyles [7,16]. While efforts have been made to address unhealthy diets by promoting actions such as reducing salt intake and advocating for polyunsaturated fats through mass media campaigns, policy actions to mitigate dietary risks remain fragmented and difficult to track [23,24]. Despite these initiatives, there are notable gaps in the formulation and implementation of comprehensive NCD policies in Cameroon, a challenge shared by many Sub-Saharan African nations [25]. The mid-point evaluation of the WHO GAP for the Prevention and Control of Non-Communicable Diseases 2013–2020 indicates progress in certain areas across 194 countries, such as establishing NCD units and implementing NCD risk factor policies [26]. However, overall performance remains deficient in areas like NCD policies, research policies, monitoring systems, and national coordination mechanisms. Consequently, there has been little progress in developing norms and standards for NCD management through primary care strategies.

Regarding interventions, the Commission identified 123 priority health interventions for implementation and scale-up in Cameroon’s healthcare system by integrating them into existing platforms for chronic diseases, such as HIV/AIDS, maternal, neonatal, and child health, and surgical services. This recommendation was inspired by lessons from Cambodia and other countries, which showed promising results when NCDI services were integrated with existing HIV treatment platforms [27]. In Cambodia, for instance, this integrated approach combined hypertension and diabetes services with Antiretroviral Therapy (ART) clinics [4,28,29]. In South Africa, the integrated service delivery model that combines HIV, Tuberculosis (TB), and NCD care has proved crucial in rural settings and is now helping the country in its post-COVID-19 health system recovery, as well as preparing it for future pandemics by moving away from isolated care. This approach of merging infectious and NCD services may allow more individuals to be screened, treated, and retained for a broader range of health conditions [27–29], which, in turn, may offer a sustainable solution for managing both infectious and non-communicable diseases.

Although WHO recognises integrated strategies as cost-effective ‘best buys’ [30], implementing integrated delivery models remains a challenge for many health systems. This is partly because integration requires expanding key health system elements, including infrastructure, staffing, training, guidelines, medications, equipment, diagnostics, and referral networks. While many of these elements are present in public health facilities at a basic level, their availability and preparedness can vary significantly. For instance, in Ethiopia, challenges to scaling up included capacity constraints such as underdeveloped primary healthcare infrastructure, shortages of healthcare workers, essential medicines, and diagnostic equipment [31]. Despite this challenge, countries would need to make the necessary efforts to integrate health services delivery. This is because studies from Ethiopia and Kenya suggest that integration could increase life expectancy at birth and avert approximately 41,000 and 9300 premature deaths by 2030, respectively [31,32]. Also, as fiscal space tightens, integration can improve efficiency, reduce donor dependence, eliminate financial waste, and enable governments to build more resilient healthcare systems [33].

One main limitation of this study is that the commission did not assess the costs of implementing prioritised interventions as a factor for selection, unlike other LMICs such as Kenya, Ethiopia, and Nepal, which considered financial implications in their prioritisation criteria [31,32,34] An important next step will be to translate this prioritised package into an operational roadmap through intervention costing, fiscal space analysis, and phased sequencing based on affordability, workforce requirements, and supply chain capacity. We strongly recommend that the costing of these interventions be considered during Cameroon’s UHC agenda implementation phase [35]. Second, the analysis relied heavily on secondary data from the GBD database and published literature, which may not fully reflect local variations in disease burden, health-seeking behaviours, or service readiness. The absence of recent, nationally representative NCDI surveillance data limited the ability to validate findings with locally generated evidence. Also, observed increases in absolute NCDI burden between 1990 and 2019 reflect a combination of epidemiologic transition, population growth, and changes in age structure. As this analysis prioritises absolute burden to inform service delivery and resource planning, it does not fully disentangle changes in disease rates from demographic effects. Future analyses incorporating age-standardised trends and subnational data would further strengthen the interpretation of temporal patterns. Lastly, the 100 participant threshold applied to quantitative studies may bias the evidence base toward more common conditions and urban facility–based studies; however, findings from the literature review were triangulated with GBD estimates to mitigate this limitation.

## CONCLUSIONS

The Cameroon NCDI Poverty Commission was established to evaluate the NCDI burden and prioritise interventions aligned with national health strategies to achieve UHC. Findings from this evaluation highlight a growing NCDI burden overshadowed by infectious diseases. The commission identified 49 priority conditions and suggested 123 evidence-based interventions for implementation. Despite the need for significant financial investment, urgent action is crucial given the substantial burden of NCDIs and deficiencies in the country’s healthcare system. Insufficient reliable data calls for a comprehensive strategy promptly. By tailoring interventions and improving data collection, Cameroon can reduce the impact of NCDIs. The first recommendation is to boost NCDI service delivery through evidence-based packages and decentralised services. The second is to enhance NCDI financing by advocating for increased government funding, seeking external support, and implementing strategies to reduce out-of-pocket expenses. The third is to strengthen data systems by updating platforms, improving data management, and conducting additional surveys. The fourth is to enhance policy development through cross-sector collaboration and regulatory improvements. The final recommendation is to improve NCDI research by prioritising initiatives for rural populations with high costs and integrating NCDI conditions into surveys for better research outcomes.

## Additional material


Online Supplementary Document

## Data Availability

**Data availability:** Data from the corresponding author of this research is available upon reasonable request.

## References

[1] Institute for Health Metrics and Evaluation (IHME). Global Burden of Disease (GBD). 2019. Available: https://www.healthdata.org/research-analysis/gbd. Accessed: 13 October 2025.

[2] Bukhman G Mocumbi AO Atun R Becker AE Bhutta Z Binagwaho A The Lancet NCDI Poverty Commission: bridging a gap in universal health coverage for the poorest billion. Lancet. 2020;396:991–1044. 10.1016/S0140-6736(20)31907-3 32941823 PMC7489932

[3] Niessen LW Mohan D Akuoku JK Mirelman AJ Ahmed S Koehlmoos TP Tackling socioeconomic inequalities and non-communicable diseases in low-income and middle-income countries under the Sustainable Development agenda. Lancet. 2018;391:2036–46. 10.1016/S0140-6736(18)30482-3 29627160

[4] Jan S Laba TL Essue BM Gheorghe A Muhunthan J Engelgau M Action to address the household economic burden of non-communicable diseases. Lancet. 2018;391:2047–58. 10.1016/S0140-6736(18)30323-4 29627161

[5] National Commissions. NCDI Poverty Network. 2025. Available: https://www.ncdipoverty.org/national-commissions-1. Accessed: 13 October 2025.

[6] Gupta N Mocumbi A Arwal SH Jain Y Haileamlak AM Memirie ST Prioritizing Health-Sector Interventions for Noncommunicable Diseases and Injuries in Low- and Lower-Middle Income Countries: National NCDI Poverty Commissions. Glob Health Sci Pract. 2021;9:626–639. 10.9745/GHSP-D-21-00035 34593586 PMC8514044

[7] Mapa-Tassou C, Bonono CR, Assah F, Ongolo-Zogo P, Sobngwi E, Mbanya JC. Analysis of non-communicable diseases prevention policies in Cameroon: Final report. 2017. Available: http://hdl.handle.net/10625/57550. Accessed: 13 October 2025.

[8] Ministry of Public Health. Cameroon. Health Sector Strategy 2016-2027. Available: https://www.minsante.cm/site/sites/default/files/HSS_english_0.pdf. Accessed: 13 October 2025.

[9] Enquête Camerounaise Auprès des Ménages. 5th round (ECAM 5): Key Indicators - Institut National de la Statistique du Cameroun. 2024. Available: https://ins-cameroun.cm/en/statistique/ecam-5-key-indicators/. Accessed: 13 October 2025.

[10] World Bank Open Data. World Bank Open Data. 2023. Available: https://data.worldbank.org. Accessed: 13 October 2025.

[11] Rudan I Song P Adeloye D Campbell H Journal of Global Health’s Guidelines for Reporting Analyses of Big Data Repositories Open to the Public (GRABDROP): preventing ‘paper mills’, duplicate publications, misuse of statistical inference, and inappropriate use of artificial intelligence. J Glob Health. 2025;15:01004. 10.7189/jogh.15.01004 40587200 PMC12208284

[12] World Health Organisation. Global Health Expenditure Database. 2023. Available: https://apps.who.int/nha/database. Accessed: 13 October 2025.

[13] FairChoices – DCP Analytics Tool. 2023. Available: https://fairchoices.w.uib.no/. Accessed: 13 October 2025.

[14] Nde CJ Raymond A Saidu Y Reaching Universal Health Coverage by 2035: Is Cameroon on Track? Univers J Public Health. 2019;7:110–7. 10.13189/ujph.2019.070304

[15] Nde CJ Raymond A Cheng NI Nzuobontane D Atemnkeng JT Mbacham WF Progress towards Universal Health Coverage: Is Cameroon Investing Enough in Primary Care? Univers J Public Health. 2019;7:171–8. 10.13189/ujph.2019.070403

[16] Haileamlak A The Impact of COVID-19 on Non-Communicable Diseases. Ethiop J Health Sci. 2022;32:1–2. 35250211 10.4314/ejhs.v32i1.1 PMC8864391

[17] World Health Organization. Noncommunicable diseases: Risk factors and conditions. Available: https://www.who.int/data/gho/data/themes/topics/topic-details/GHO/ncd-risk-factors. Accessed: 13 October 2025.

[18] United Nations. Country Insights. Available: https://hdr.undp.org/data-center/country-insights. Accessed: 13 October 2025.

[19] International Monetary Fund. African Department. Climate Change in Cameroon: Key Challenges and Reform Priorities. 2024. Available: https://www.elibrary.imf.org/view/journals/002/2024/052/article-A003-en.xml. Accessed: 13 October 2025.

[20] Fezeu L Kengne AP Balkau B Awah PK Mbanya JC Ten-year change in blood pressure levels and prevalence of hypertension in urban and rural Cameroon. J Epidemiol Community Health. 2010;64:360–5. 10.1136/jech.2008.086355 19692732 PMC3094348

[21] Africa’s Health Financing in a New Era – April. Africa CDC. 2025. Available: https://africacdc.org/news-item/africas-health-financing-in-a-new-era-april-2025/. Accessed: 13 October 2025.

[22] World Health Organization. Global action plan for the prevention and control of noncommunicable diseases 2013-2020. Available: https://www.who.int/publications/i/item/9789241506236. Accessed: 13 October 2025.

[23] StopBlaBlaCam. 30% of Cameroonians have high blood pressure, mostly due to excessive salt consumption. Available: https://www.stopblablacam.com/society/1603-6215-30-of-cameroonians-have-high-blood-pressure-mostly-due-to-excessive-salt-consumption. Accessed: 5 November 2025.

[24] Aminde LN Cobiac L Veerman JL Cost-effectiveness analysis of population salt reduction interventions to prevent cardiovascular disease in Cameroon: mathematical modelling study. BMJ Open. 2020;10:e041346. 10.1136/bmjopen-2020-041346 33234652 PMC7689085

[25] Kassa MD Grace JM Noncommunicable Diseases Prevention Policies and Their Implementation in Africa: A Systematic Review. Public Health Rev. 2022;42:1604310. 10.3389/phrs.2021.1604310 35295954 PMC8865333

[26] World Health Organization. Mid-point evaluation of the implementation of the WHO global action plan for the prevention and control of noncommunicable diseases 2013–2020 (NCD-GAP). Volume 1: Report. Geneva, Switzerland: World Health Organization; 2020. Available: https://www.who.int/publications/m/item/mid-point-evaluation-of-the-implementation-of-the-who-global-action-plan-for-the-prevention-and-control-of-noncommunicable-diseases-2013-2020-(ncd-gap). Accessed: 1 October 2026.

[27] Janssens B Van Damme W Raleigh B Gupta J Khem S Soy Ty K Offering integrated care for HIV/AIDS, diabetes and hypertension within chronic disease clinics in Cambodia. Bull World Health Organ. 2007;85:880–5. 10.2471/BLT.06.036574 18038079 PMC2636263

[28] Solanki G Kelly G Cornell J Geffen L Doherty T The need to incorporate the impact of population ageing into the post-COVID-19 policy and planning reset in Low and Middle Income Countries. Glob Health Action. 2021;14:1921351. 10.1080/16549716.2021.1921351 34013832 PMC8158212

[29] Topp SM Chipukuma JM Giganti M Mwango LK Chiko LM Tambatamba-Chapula B Strengthening Health Systems at Facility-Level: Feasibility of Integrating Antiretroviral Therapy into Primary Health Care Services in Lusaka, Zambia. PLoS One. 2010;5:e11522. 10.1371/journal.pone.0011522 20644629 PMC2903482

[30] World Health Organization; World Economic Forum. From Burden to ‘Best Buys’: Reducing the Economic Impact of Non-Communicable Disease in Low and Middle-Income Countries. Geneva, Switzerland: World Health Organization; 2011. Available: https://cdn.who.int/media/docs/default-source/medical-devices/health-technology-assessment/from-burden-to-best-buys-reducing-the-economic-impact-of-ncds-in-low-and-middle-income-countries.pdf. Accessed: 1 October 2026.

[31] Memirie ST Dagnaw WW Habtemariam MK Bekele A Yadeta D Bekele A Addressing the Impact of Noncommunicable Diseases and Injuries (NCDIs) in Ethiopia: Findings and Recommendations from the Ethiopia NCDI Commission. Ethiop J Health Sci. 2022;32:161–80. 35250228 10.4314/ejhs.v32i1.18 PMC8864405

[32] Mwangi K Gathecha G Nyamongo M Kimaiyo S Kamano J Bukachi F Reframing Non-Communicable Diseases and Injuries for Equity in the Era of Universal Health Coverage: Findings and Recommendations from the Kenya NCDI Poverty Commission. Ann Glob Health. 2021;87:3. 10.5334/aogh.3085 33505862 PMC7792462

[33] World Health Organization. WHO issues guidance to address drastic global health financing cuts. 2025. Available: https://www.who.int/news/item/03-11-2025-who-issues-guidance-to-address-drastic-global-health-financing-cuts. Accessed: 5 November 2025.

[34] Koirala B Adhikari SR Shrestha A Vaidya A Aryal KK Kalaunee SP A National Equity Initiative to Address Noncommunicable Diseases and Injuries: Findings and Recommendation from the Nepal NCDI Poverty Commission. Kathmandu Univ Med J KUMJ. 2022;20:376–83. 10.3126/kumj.v20i3.54013 37042383

[35] Pholpark A Mon HM Lim LT Tsilaajav T de Oliveira Cruz V Hanvoravongchai P Challenging inadequate and fragmented primary health care financing: findings from Bangladesh, Indonesia, Maldives, and Nepal. Lancet Reg Health - Southeast Asia. 2025;38:100613. 10.1016/j.lansea.2025.100613 PMC12173036 40528875

