# Supplementary material for: Addressing non-communicable diseases and injuries through an equity lens: insights from the Cameroon non-communicable disease and injury (NCDI) Poverty Commission
Source: J Glob Health. 2026 Oct 9;16:04266. doi: 10.7189/jogh.16.04266 (PMC13651764; doi:10.7189/jogh.16.04266)
Supplement: Supplementary file 1 [file jogh-16-04266-s001.pdf]

Suppement to: Saidu Y, Budzi M, Ben Bachire H, Ousmane D, Haoua F, Clarisse M, Emmanuel V, Ndom MS, Mbanga C, Karimu I, Sonyuy MF, Mensah E, Bukhman G, Gupta N, Njei B, Al Ajlouni YA, Fasawe O, Sap S, Sobngwi E, Ndom P, Kingue S. Addressing non-communicable diseases and injuries through an equity lens: insights from the Cameroon non-communicable disease and injury (NCDI) Poverty Commission. J Glob Health. 2026;16:04266.

Adherence to JoGH's Guidelines for Reporting Analyses of Big Data Repositories Open to the Public (GRABDROP)

Manuscript: "Addressing non-communicable diseases and injuries through an equity lens: insights from the Cameroon non-communicable disease and injury (NCDI) Poverty Commission." The table below addresses each of the five GRABDROP items in relation to this manuscript, which is based on secondary analysis of the Global Burden of Disease (GBD) 2019 database together with a literature review and national health-financing data.

GRABDROP guideline item	Author response / declaration
**1.** Please list all papers published by each co-author in the previous three years that were based on secondary analysis of a big data repository.	This manuscript is itself a secondary analysis of a publicly available big-data repository (the Global Burden of Disease [GBD] 2019 database), combined with a systematic literature review and national health-financing data. It was produced by a multidisciplinary National NCDI Poverty Commission of experts from government, academia, clinical practice, and civil society, working with the NCDI Poverty Network at Brigham & Women's Hospital. All listed authors made substantive intellectual contributions, as detailed in the Author Contributions statement, and the work does not originate from a 'paper mill.'<br><br>Among the co-authors, three are contributors to secondary analyses of public big-data repositories, principally as members of the GBD Collaborator Network. Notably, three co-authors of the present manuscript (B Njei, Y A Al-Ajlouni, and Y Saidu) recently co-authored a GBD secondary analysis of mental health in sub-Saharan Africa:<br><br>Njei B, Al-Ajlouni YA, Lemos SY, Motaze NV, Cleenewerck de Kiev L, Bain LE, Saidu Y. Over three decades of mental health in sub-Saharan Africa: an analysis of GBD data from 1990 to 2023. Glob Health Epidemiol Genom. 2026. doi:10.1155/ghe3/5570226<br><br>Further representative works from the previous three years are:<br><br>**Basile Njei:**<br><br>GBD 2023 Diarrhoeal Disease and Enteric Infectious Diseases Collaborators. Global burden of enteric infectious diseases, diarrhoeal diseases, and corresponding aetiologies, 1990–2023: a systematic analysis for the GBD Study 2023. Lancet Infect Dis. 2026 (named collaborator). doi:10.1016/S1473-3099(26)00194-5<br><br>Lean MASLD in US adults: undiagnosed burden and metabolic features from NHANES 2017–2020. J Gen Intern Med. 2025. doi:10.1007/s11606-025-10086-8 (with Y A Al-Ajlouni)<br><br>Additional analyses of GBD, NHANES, and US National/Nationwide Inpatient Sample data, including ACG conference abstracts<br><br>**Yazan A Al-Ajlouni:**

GRABDROP guideline item	Author response / declaration
	GBD 2021 Forecasting Collaborators. Burden of disease scenarios for 204 countries and territories, 2022–2050: a forecasting analysis for the GBD Study 2021. Lancet. 2024 (named collaborator). doi:10.1016/S0140-6736(24)00685-8<br>Named collaborator on GBD 2023 systematic analyses, Lancet 2025 (e.g. Burden of 375 diseases and injuries..., doi:10.1016/S0140-6736(25)01637-X).<br>NHANES lean-MASLD analysis, J Gen Intern Med 2025
**2.** Please explain the key elements of your study design and the use of the available datasets that make your study an original scientific contribution.<br>.	This study is an original scientific contribution and not a repackaging of previously published GBD outputs. Rather than extracting and re-presenting estimates for a single disease or risk factor, the Commission:<br>(i) triangulated multiple data sources — GBD 2019 estimates, a comprehensive review of 391 peer-reviewed articles and grey literature (2005–2022), and national health-financing data from the WHO Global Health Expenditure Database and national planning documents;<br>(ii) applied an original two-phase priority-setting methodology, scoring NCDI conditions across four quantitative criteria (burden, severity, disability, and equity) followed by structured qualitative review and expert consensus; and<br>(iii) used the FairChoices DCP Analytics Tool, parameterised with Cameroon-specific financing inputs, to model feasible intervention packages under realistic budget constraints.<br>These methods generated novel, policy-relevant outputs not previously available for Cameroon: a ranked set of 49 priority NCDI conditions and 123 prioritised interventions mapped to specific health-system delivery platforms (community, health centre, district, and reference hospital), together with an analysis of NCDI financing gaps. The study addresses a new research question in a Central African setting where such data are scarce.
**3.** Please list all publications that addressed similar research questions in the same dataset and indicate where you cited them in your paper.	A comprehensive literature review (391 peer-reviewed articles plus grey literature) was conducted to identify existing evidence on NCDIs in Cameroon, and relevant national and GBD-informed studies were cited in the Introduction, Methods, and Discussion. While numerous global, regional, and country-level analyses of GBD 2019 NCDI data exist, and NCDI Poverty Commission analyses have been published for other countries (cited as references 2 and 6), to the authors' knowledge no prior study has combined GBD 2019 estimates with a national literature review, a health-financing analysis,

GRABDROP guideline item	Author response / declaration
	and FairChoices intervention modelling to conduct an NCDI Poverty Commission priority-setting exercise specific to Cameroon.<br>The authors are not aware of any other group testing the same research question using the same dataset and methods for Cameroon.
**4.** Please explain how you addressed multiple testing through an appropriately rigorous statistical threshold and indicate this in the methods section.	The concern regarding false-positive associations arising from multiple hypothesis testing does not apply to this study, because no inferential hypothesis testing was performed. The analysis of GBD 2019 data was descriptive: cause-specific mortality and morbidity were summarised as point estimates, proportions, and DALY distributions by disease category and age group, and temporal trends (1990–2019) were described.<br>No p-values, significance thresholds, or association tests were computed; therefore no correction for multiple comparisons (e.g. Bonferroni) was required. All GBD estimates are reported with the 95% uncertainty intervals provided by IHME, and point estimates (mean values) are reported throughout. This is stated in the Methods section ('GBD 2019 data extraction and analysis').
**5.** Please declare to what extent AI chatbots have been used in developing your paper and to which parts of the paper they contributed.	AI-based tools were used solely for language editing specifically to check English grammar and improve the readability of author-drafted text. AI chatbots / large language models were not used to conceive the study, to extract or analyse data, to generate results, tables, or figures, to interpret findings, or to draft the scientific content.<br>All data analysis, interpretation, and conclusions are the work of the authors, who take full responsibility for the accuracy and integrity of the content, consistent with JoGH's policy on the use of AI in scholarly manuscripts.

GBD – Global Burden of Disease; DALY – disability-adjusted life year; IHME – Institute for Health Metrics and Evaluation; JoGH – Journal of Global Health; NCDI – non-communicable disease and injury; UHC – Universal Health Coverage.
